# Comparative genomics of *Plasmodium yoelii nigeriensis* N67 and N67C: genome-wide polymorphisms, differential gene expression, and drug resistance

**DOI:** 10.1186/s12864-024-10961-4

**Published:** 2024-11-05

**Authors:** Jian Wu, Cihan Oguz, Awet Alem Teklemichael, Fangzheng Xu, Rachel V Stadler, Amuza Byaruhanga Lucky, Shengfa Liu, Osamu Kaneko, Justin Lack, Xin-zhuan Su

**Affiliations:** 1 Malaria Functional Genomics Section, Laboratory of Malaria and Vector Research, National Institute of Allergy and Infectious Disease, National Institutes of Health, Rockville, MD 20852 USA; 2 Integrated Data Sciences Section, Research Technologies Branch, National Institute of Allergy and Infectious Diseases, National Institutes of Health, Bethesda, MD 20892 USA; 3 Department of Protozoology, Institute of Tropical Medicine (NEKKEN), Nagasaki University, Nagasaki, 852-8523 Japan; 4 State Key Laboratory of Cellular Stress Biology, School of Life Sciences, Faculty of Medicine and Life Sciences, Xiamen University, Xiamen, Fujian 361102 China

**Keywords:** *Plasmodium*, Mouse, Genome sequencing, Proteome, Polymorphism, Differentially expressed genes (DEGs)

## Abstract

**Background:**

The study of rodent malaria parasites has significantly advanced our understanding of malaria parasite biology and host responses to parasite infections. There are four well-characterized rodent malaria parasite species (*Plasmodium yoelii, P. chabaudi, P. berghei,* and *P. vinckei*). Each species also has multiple strains that cause different disease phenotypes. *P. yoelii nigeriensis* N67C and N67, two isogenic parasites, are particularly intriguing as they differ in virulence and incite different immune responses in mice. The genome of the N67 parasite has been assembled recently, but not that of N67C. This study used PacBio HiFi sequencing data to assemble the N67C genome, compared the two genomes, and performed RNA sequencing to identify polymorphisms and differentially expressed genes (DEGs).

**Results:**

The assembled N67C parasite genome consisted of 16 scaffolds and three contigs of approximately 22.5 Mb with 100% and 96.6% completeness based on well-characterized single-copy orthologs specific to the Apicomplexa phylum and the *Plasmodium* genus, respectively. A comparison between the annotated N67C and N67 genomes revealed 133 single nucleotide polymorphisms (SNPs) and 75 indels. Among the polymorphic sites, an S (N67) to N (N67C) amino acid substitution at position 114 (S114N) in the dihydrofolate reductase-thymidylate synthase (DHFR-TS) confers resistance to pyrimethamine in mice. Additionally, 60 differentially expressed single-copy genes (DEGs) were detected after comparing mRNA levels between the two parasites. Starting with the predicted and annotated 5,681 N67C and 5,749 N67 genes, we identified 4,641 orthogroups that included at least one gene from the four *P. yoelii* parasites (N67, N67C, 17X, and YM), whereas 758 orthogroups showed subspecies or strain-specific patterns.

**Conclusion:**

The identification of polymorphic sites between the N67 and N67C genomes, along with the detection of the DEGs, may provide crucial insights into the variations in parasite drug responses and disease severity between these two isogenic parasites. The functional characterization of these genetic differences and candidate genes will deepen our understanding of disease mechanisms and pave the way for developing more effective control measures against malaria.

**Supplementary Information:**

The online version contains supplementary material available at 10.1186/s12864-024-10961-4.

## Background

Malaria is a deadly disease caused by *Plasmodium* parasites that still infects hundreds of millions of people and kills over half a million a year [1]. The lack of a complete understanding of parasite biology and disease mechanisms has impeded disease control and elimination, including the development of a sterile vaccine that can prevent parasite infection. The complicated two-host parasite life cycle, ethical regulations, and high costs have prevented in-depth studies of disease molecular mechanisms in humans directly. Parasites infecting rodents and non-human primates have been widely used for drug and vaccine development and studying host-parasite interaction [2–4]. Given that the transmission of the parasites to *Anopheles* mosquitoes could be easily achieved in a laboratory, animal models of malaria parasites have contributed greatly to studying various biological aspects of malaria parasites, especially the development of pre-erythrocytic and sexual stages and host response to parasite infections [5, 6].

There are four rodent malaria parasite species (*Plasmodium yoelii, Plasmodium chabaudi, Plasmodium berghei,* and *Plasmodium vinckei*) commonly used to study parasite genetics, parasite development, mechanisms of host-parasite interaction, and vaccine evaluation [7, 8]. The rodent parasites were initially isolated from the African thicket rats and have been adapted to grow in laboratory mice. *P. yoelii* has four subspecies: *P. yoelii yoelii, P. y. killicki, P. y. nigeriensis* as well as *P. y. cameronensis* that was recently characterized [7, 9]. *P. y. yoelii* strains such as 17X, 17XNL, 17XL, YM, 33X, By265 as well as subspecies *P. y. nigeriensis* N67 and N67C have been widely used in various genetic studies [7, 10]. Among the *P. yoelii* strains, 17X, 17XNL, 17XL, and YM are isogenic strains; in fact, 17XNL, 17XL, and YM were derived from the 17X strain during propagation in different laboratories [11]. In a recent study, 1,955 variant sites were identified between the 17X and 17XNL genomes, although most polymorphic sites were small indels in intergene regions [12]. Similarly, genotyping analyses suggested that N67, N67C (or 33X(Pr3)), Py-Kenya, Py-NS, and Py-238y were closely related or possibly derived from a common *P. yoelii* ancestor [13–15]. For example, only 22 single nucleotide polymorphisms (SNPs) were detected between N67 and N67C after the hybridization of N67 and N67C DNAs to an SNP microarray containing ~ 11,000 probes [15], suggesting that N67 and N67C parasite genomes diverged relatively recently.

Among the isogenic parasites, 17X and 17XNL are non-lethal, whereas 17XL and YM kill C57BL/6 mice about seven days after injecting 1 × 10^6^ infected red blood cells (iRBCs) [16]. Parasites N67 and N67C are both lethal; N67C kills C57BL/6 mice in approximately seven days, whereas N67 kills its host by day 20 post-infection (pi) [17, 18]. N67 infection stimulates an early type I interferon (IFN-I) response (18–24 h pi) that was linked to the suppression of parasitemia on day 7 pi, whereas N67C infection causes extensive inflammation with a high level of interferon-gamma (IFN-ℽ) [17–19]. A C741Y substitution in the trafficking domain of *P. yoelii* erythrocyte binding-like protein (PyEBL) was shown to contribute to the differences in growth-related virulence and IFN-I levels between N67 and N67C [14, 20]. Additionally, a genetic locus at one end of chromosome 13 was significantly linked to the expression of many interferon-stimulated genes (ISGs) after quantitative trait loci (QTL) analysis of progeny from an N67 × 17XNL cross [19]. Comparison of the N67 and N67C genome sequences and thorough categorization of the genetic differences between the two parasites may reveal additional functional genetic polymorphisms that modulate virulence and host-parasite interaction. Here, we performed genome and RNA sequencing (RNA-Seq) using the PacBio and Illumina sequencing technologies, respectively. We then annotated the parasite proteome and compared the N67C genome sequences to those of N67 reported previously [9, 15, 21] to reveal potential functional polymorphisms and differentially expressed genes (DEGs) that may play essential roles in virulence and host-parasite interaction.

## Results

### PacBio sequencing and the assembly of the N67C parasite genomes

**Fig. 1:**
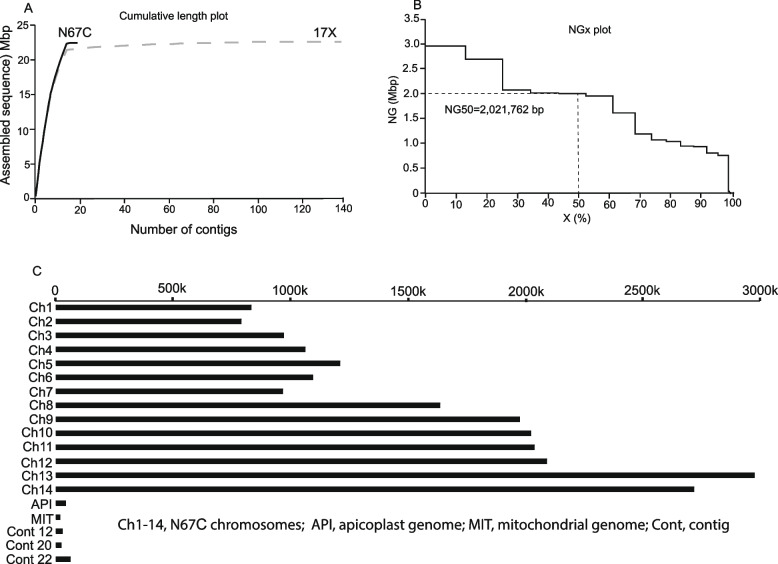
Statistics and graphic displays of de novo assembled *Plasmodium y. nigeriensis* N67C chromosomes and contigs. **A** Plots of cumulative genome size in megabase pair (Mbp) versus the number of contigs for the *P. y. nigeriensis* N67C (solid line) and *P. y. yoelii* 17X (dash line). **B** Plots of the assembled contig size as a function of the percentage of contigs (x or NGx plot.), with x ranging from 0 to 100%. **C** Graphic presentation of the *P. y. nigeriensis* N67C chromosomes and the genomes of apicoplast (API) and mitochondrion (MIT). The scale line on the top indicates the sizes of the chromosomes

After filtering procedures to remove barcodes and low-quality sequences from the PacBio sequencing, the N67C genome was assembled using the Flye algorithm [21, 22]. The DNA sequences of the N67C parasite were de novo assembled into 19 contigs consisting of a total length of 22,453,501 bp (Table 1), with the largest assembled sequence being 2,976,465 bp having an average GC content of 21.9%. A plot of sequence length (Mbp) against the number of contigs showed that the N67C genome reached more basepairs than those of published 17X assembly after 13 contigs (Fig. 1A) with an NG50 value equal to 2,021,762 bp (Fig. 1B). The assembled contigs included the parasite’s 14 chromosomes and the genomes of apicoplast and mitochondrion (Fig. 1C). The largest chromosome is chromosome 13 having 2,976,465 bp and the smallest chromosome is chromosome 2 with 780,425 bp (Fig. 1C and Table 1). The mitochondrial genome has 5,957 bp, and the apicoplast genome has 34,317 bp. There were also three contigs, approximately 17.4 kb, 16.3 kb, and 51.9 kb, respectively, that were not assigned to any chromosome (Table 1). The sequences of chromosomes 1–14 plus the mitochondrial and the apicoplast genomes account for 99.62% of the N67C assembly.

**Table 1:** Assembled chromosomes and contigs from *Plasmodium y. nigeriensis* N67C parasite

N67C chromosome	Seq_length (bp)	17X chromosome
1	827,609	Py17X_01_v3
2	780,425	Py17X_02_v3
3	963,616	Py17X_03_v3
4	1,056,810	Py17X_04_v3
5	1,207,055	Py17X_05_v3
6	1,089,878	Py17X_06_v3
7	960,445	Py17X_07_v3
8	1,632,432	Py17X_08_v3
9	1,970,802	Py17X_09_v3
10	2,021,762	Py17X_10_v3
11	2,035,634	Py17X_11_v3
12	2,089,434	Py17X_12_v3
13	2,976,465	Py17X_13_v3
14	2,715,216	Py17X_14_v3
Plastid genome	34,317	Py17X_API_v3
Mitochondrial genome	5,957	Py17X_MIT_v3
Contig 12, Unassigned	17,424	
Contig 20, Unassigned	16,292	
Contig 22, Unassigned	51,928	
	22,453,501

**Fig. 2:**
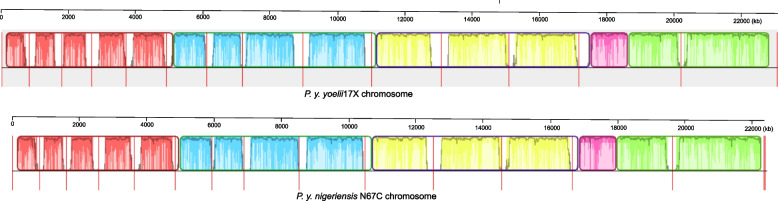
Alignment of assembled N67C contigs to the 17X chromosomes. The alignments were generated using progressiveMauve which uses a positional homology approach to compare the 17X genome and the N67C assemblies scaffolded. Each color corresponds to a locally collinear block (LCB) that is conserved across the two genomes. Inside each LCB, the jagged dark lines represent the similarity profile. The height of the similarity profile at a given point corresponds to the average level of conservation in that region of the genome sequence. The profile's height was calculated using an entropy model that measured deviation from randomness. The higher the profile at a given point, the more that region of the chromosome deviates from the similarity expected by random chance. Overall, the height of the similarity profile was calculated to be inversely proportional to the average alignment column entropy over a region of the alignment. Therefore, a sudden drop in the profile (a "valley") indicates a region where the similarity score between the two sequences is low. For each region, the shaded & jagged areas show the range of similarity values. The vertical red lines indicate chromosome boundaries. The regions outside the LCBs (white regions) lack detectable homology among the pair of genomes compared. Areas within the blocks that are entirely white do not align with the other

The sequences (contig size ≥ 500 bp) were also assembled based on the reference 17X genome using the scaffolding tool RaGOO (ragtag.scaffold), producing 138 contigs consisting of 22,592,200 bp. Of the sequences, 21,237,836 bp (94.0%) were aligned to the 17X genome (Fig. 2), with the largest aligned segment of 693,555 bp and the size of the smallest contig that makes up 50% of the genome being (NG50) 202,1762 bp. There was also one unaligned contig of 16,292 bp and 16 partially aligned contigs of 1,040,699 bp. The large numbers of partially aligned contigs reflect the divergence of the N67C and 17X parasites that belong to two subspecies (*P. y. yoelii* 17X and *P. y. nigeriensis* N67C).

To evaluate the completeness of the genome assembly and annotation, we calculated the coverage of benchmarking universal single-copy orthologs (BUSCOs) that can quantitatively assess the completeness of genome assembly based on evolutionarily informed expectations of gene content from near-universal single-copy orthologs [23]. The assembled N67C genome had 100% (446 out of 446) BUSCO-based completeness levels of Apicomplexan single-copy orthologs and 96.6% (3,517 out of 3,642) BUSCO of *Plasmodium* single-copy orthologs with a 664X mean coverage.

### RNA-Seq and gene models from the N67C parasite genome

**Fig. 3:**
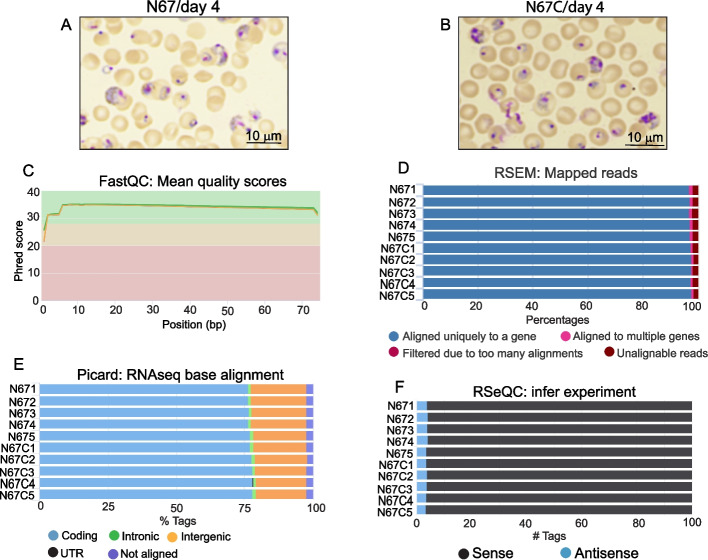
Characterization of cDNA reads of mixed blood stages of *Plasmodium y. nigeriensis* N67 and N67C. mRNAs were extracted from blood samples with mixed stages of parasites collected from 10 mice, five infected with either N67 or N67C parasites, and were processed for directional RNA-Seq as described in the Methods. **A** and** B** Representative images of blood smears from mice infected with N67 and N67C day 4 post-infection. **C** Mean quality scores of cDNA reads estimated using FASTQC [26]. Mean Phred quality score plot against each base call position. **D** Percentages of reads mapped to a gene or multiple gene families using RSEM package [27]. **E** Percentages of reads mapped to coding, intronic, intergenic, untranslated regions (UTR), or not aligned using Picard [25]. **F** Percentages of reads from sense and antisense transcripts predicted using RSeQC [24]

We compared the expression profiles of the mixed blood stages of N67 and N67C parasites with similar proportions of ring, trophozoite, and schizont stages (Fig. 3A and B) using RNA-Seq data derived from five mice (for each parasite strain), each contributing to a distinct cDNA library (five replicates for each parasite). RNA-Seq quality and the expression levels were assessed using RSeQC [24], Picard [25], and FastQC [26], and RSEM [27]. For each sample, 21.3 to 31.1 million reads were mapped to the assembled N67C genome with an average read length of 73 bp and mean coverage value ranging from 138.6X to 228.4X (Table S1). The read quality had a Phred score > 30 over the entire read length (Fig. 3C). Among the mapped reads, more than 95% were aligned uniquely to a gene (Fig. 3D), and > 75% of the reads were mapped to coding regions (Fig. 3E). Approximately 97% of reads were from sense mRNA (Fig. 3F).

**Fig. 4:**
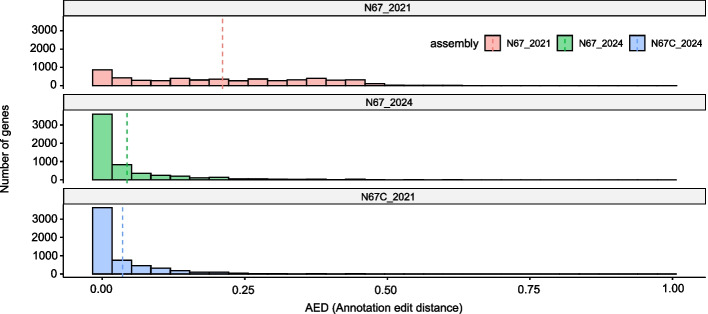
Plots of the average annotation edit distances (AEDs) for the three annotated genomes. The N67C_2024 and N67_2024 assemblies were generated using Flye, and the N67_2021 assembly was generated using HGAP. The vertical dashed lines represent the average AED values of 0.03579, 0.04357, and 0.2109 for the N67C_2024, N67_2024, and N67_2021 assemblies

Next, we used MAKER to annotate the assembled N67/N67C genomes with the 17X proteome and gene models available in the public databases to predict genes and proteins for the N67C genome. We obtained 5,681 predicted genes/proteins from the N67C genome with good statistical support (Table S2). Gene annotation quality was quantified using annotation edit distance (AED) that reflects the difference between the predicted sequences of the N67C and the well-characterized 17X genes. AED values range between 0 and 1, with lower AED values indicating higher annotation quality and higher AED values pointing to weaker support for annotation. For a well-annotated genome, the AED is expected to be less than 0.5 for at least 90% of the predicted genes [28]. Among the 5,681 predicted N67C genes, 5,675 (99.9%) had AED values smaller than 0.5 (average AED = 0.03579, Fig. 4) that met a well-annotated genome criterion. The orthologs of the annotated N67C proteins across other *P. yoelii* parasites, including YM, 17X, and N67, and those of *P. falciparum* are listed in Table S2.

We also re-assembled the N67 genome and obtained 5,749 predicted genes (Table S3), which is an approximately 7% improvement over the 5,383 genes that were predicted previously [21]. Among the updated sets of N67 genes and proteins, 5,729 (99.6%) genes had an AED value less than 0.5 (Table S3), and the average AED values were improved from 0.2109 for the 2021 annotated genome to 0.04357 for the 2024 annotated genome (Fig. 4). Among the 5,749 genes, 5,424 (95.4%) genes had an ortholog in the N67C genome (Table S2).

### Differentially expressed genes (DEGs) between the N67 and N67C parasites

Next, we performed differential expression analysis of these two parasites using the RNA-Seq data from the N67 and N67C parasites. There were 317 genes that were twofold differentially expressed between N67 and N67C (N67-N67C, < log_2_[-1] and N67-N67C, > log_2_ [1]). However, most (~ 80%) of the genes encoded YIR and fam-a/b/c proteins, some of which could be due to sequence alignment errors, leaving 18 down-regulated and 42 up-regulated single-copy genes in N67C compared with N67 (Table S4). Among the 60 DEGs, 24 encoded conserved Plasmodium proteins of unknown functions. The rest of the 36 DEGs were predicted to encode proteins with diverse functions; for example, genes encoding RuvB-like helicase 1 and centrosomal protein CEP72 were the two top genes that expressed at higher levels in N67, whereas genes encoding a nucleic acid-binding protein and an AAP2 protein were the top genes expressed at higher levels in N67C. The diverse *yir/fam* gene expression patterns and increased expression of genes, such as gene encoding RuvB-like helicase 1 in the N67 parasite, may contribute to the stimulation of an early IFN-I response in mice infected with the N67 parasite [18].

Gene ontology analysis (https://geneontology.org/) of these twofold DEGs using *P. falciparum* orthologue gene IDs (Table S2) did not detect any significant enrichment. If the gene lists were extended to a 1.5-fold difference in expression, the GO biological process of microtubule-based movement was positively enriched 16-fold with FDR = 0.01 for N67. The GO-cellular components of chromosomal and centromeric regions were enriched ~ sixfold with FDR = 0.04 for the N67 parasite.

There were 503 genes that did not have detectible transcripts in N67C, most of which encoded *yoelii* interspersed repeats (YIR), fam, PYST-A, and proteins of unknown function (Table S2). Some of the genes could be expressed in liver or mosquito stages, such as sexual stage antigen s48/45 protein (N67C_002592), exported protein 1 (EXP1, or circumsporozoite-related antigen, N67C_005295), sporozoite surface protein essential for liver stage development (SPELD, N67C_003168), and liver merozoite formation protein (PALM, N67C_005307). There were also genes predicted to encode apicoplast ribosomal proteins (N67C_005671, N67C_005667, N67C_005664, N67C_005669, N67C_005668, N67C_005670), RNA polymerase Rpb1 (N67C_005663), and transcription factor TFIID (N67C_002097) that could play important roles in the development of non-blood stages (Table S2). Searches of transcriptomic data in the PlasmoDB (https://plasmodb.org/plasmo/app) showed no expression in the blood stages or all stages for some of the genes. Interestingly, the expressions of N67C_002097 (PY17X_1106900), N67C_005295 (PY17X_0101400), and N67C_005307 (PY17X_0102700) were increased in the puf2 knockout parasite [29]. Further confirmation of stage-specific expression and functional characterization of these genes may provide critical information for interrupting parasite development in the liver and mosquitoes.

### Genome-wide polymorphisms between N67 and N67C parasites

**Fig. 5:**
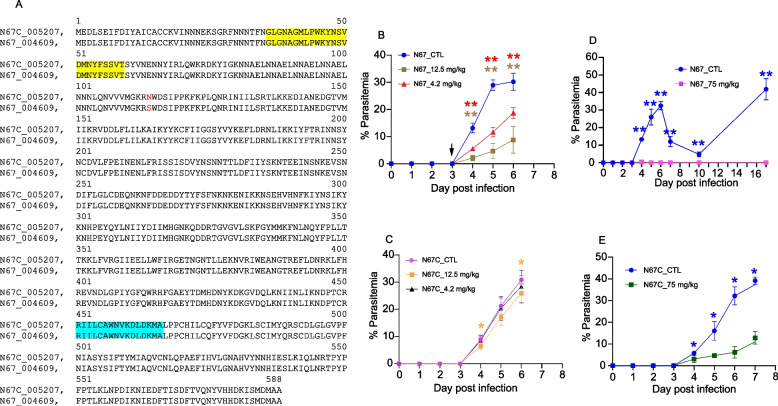
An S–N (N67-N67C) amino acid substitution in the dihydrofolate reductase-thymidylate synthase (DHFR-TS) confers resistance to pyrimethamine. **A** Sequence alignment of DHFR-TS proteins from *Plasmodium y. nigeriensis* N67 and N67C parasites. The yellow highlighted sequence is the DHFR signature domain, and the light blue highlighted sequence is the TS active site as determined by ScanProsite (https://prosite.expasy.org/cgi-bin/prosite/scanprosite/ScanView.cgi?scanfile=79818089679.scan.gz). The S to N substitution is marked in red. **B** Plots of parasitemia after treatment of N67-infected mice with two dosages of pyrimethamine (PYR). Mice were injected with 1 × 10.^6^ iRBCs. From day 3 to day 6 (arrowheads), mice were also IP-injected with PYR (4.2 mg/kg or 12.5 mg/kg) in 10% DMSO and 90% PBS). Parasitemias were counted under a microscope after Giemsa staining. **C** Plots of parasitemia after treatment of N67C-infected mice with two dosages of PYR as done in (**B**). (**D** and **E**) Plots of parasitemia in mice infected with N67 (**D**) and N67C (**E**) after treatment with a higher dosage of PYR (75 mg/kg). Note: Non-treated mice died on day 7 post-infection with N67C, whereas some treated mice survived to day 15; no parasitemia was obtained after day 7 post-infection for these two groups. Mann–Whitney U test (*n* = 5); *, *P* < 0.05; **, *P* < 0.01

One of the goals of this study is to systematically identify the genetic differences between the N67 and N67C parasites. These two parasites have similar genomes [14, 15] but produce very different disease phenotypes and stimulate dramatically different host immune responses [17, 18, 20]. The limited polymorphic sites detected between N67 and N67C using ~ 11,000 microarray probes suggested an isogenic pair of parasites [15]. Further detection of genome-wide polymorphisms between these two parasites may help identify additional genes other than the C741Y substitution in the *Pyebl* gene [20] that may contribute to various disease phenotypes and blood-stage parasite development. We, therefore, compared the N67 and N67C genomes and detected 208 polymorphic sites, including 133 SNPs and 75 indels, on the parasite 14 chromosomes and contig 12 (Table S5). Interestingly, some chromosomes are more polymorphic than others; for example, chromosome 2 is small but has 15 SNPs and nine indels between N67 and N67C, whereas the largest chromosome 13 has only three SNPs and five indels. Chromosome 11 has the most polymorphic sites with 24 SNPs and 14 indels, and chromosome 1 has only one SNP and an indel (Table 2). Among the 192 chromosomal polymorphic sites, 60 were in intergenic regions, and 33 of the 132 remaining polymorphic sites in the coding regions were within multiple gene families (genes encoding YIR or PYST-A). The known A-G (N67^A^ -N67C^G^) substitution in the erythrocyte binding-like (PyEBL) protein [14, 20] was among the coding polymorphisms. There was also a G-A (N67^G^-N67C^A^) substitution in the gene encoding bifunctional dihydrofolate reductase-thymidylate synthase (DHFR-TS), leading to an S–N (N67-N67C) amino acid substitution at position 114 in the DHFR domain (Fig. 5A). Interestingly, there are mutations in several genes encoding proteins that may bind DNA/RNA (a C2H2-type zinc finger protein, two proteins with RNA-binding domains, and a putative DNA repair endonuclease) or play a role in nucleoside metabolism (an equilibrative nucleoside transporters 1 and nucleoside triphosphate hydrolases). Changes in the proteins related to nucleic acid metabolism may affect the host recognition of parasite nucleic acids and/or nucleosides and the production of type I interferons [30].

**Table 2:** Polymorphic sites, including single nucleotide polymorphisms (SNPs) and indels between N67 and N67C parasites

Chromosome	# SNP	# Indel	Total
1	1	1	2
2	15	9	24
3	15	4	19
4	9	3	12
5	3	2	5
6	15	6	21
7	2	3	5
8	3	5	8
9	0	9	9
10	9	3	12
11	22	14	36
12	6	0	6
13	3	5	8
14	18	7	25
contig 12	12	4	16
	133	75	208

We also synthesized primers to amplify DNA segments covering the polymorphic sites in nine predicted coding regions/genes, sequenced the PCR products, and confirmed the polymorphic sites between the N67 and N67C parasite genomes (Table S6). The confirmed polymorphic sites included the A-G substitution in the PyEBL on chromosome 13 and the G-A substitution in the DHFR-TS on chromosome 7. The S114N amino acid substitution in the DHFR-TS protein may play a role in parasite response to pyrimethamine (PYR). We therefore tested the N67C and N67 parasites’ responses to PYR using Peter’s 4-day in vivo drug assay. Indeed, N67C was more resistant to PYR than N67 (Fig. 5B and C). When mice infected with N67 were treated with PYR (4.2 mg/kg or 12.5 mg/kg) for four days from day 3 pi, significant reductions in parasitemia were observed from the second day of PYR treatment (Fig. 5B). No significant change in parasitemia was observed in mice infected with N67C after treatment at 4.2 mg/kg; some reductions in parasitemia were observed after treatment with 12.5 mg/kg (significant decrease on day 2 and day 4 after PYR treatment) (Fig. 5C). If we increased the PYR dosage to 75 mg/kg, N67C parasite survived the treatment, but not N67 (Fig. 5D and E). These results confirm that the S114N amino acid substitution can confer resistance to PYR.

### Comparative genomics of four *P. yoelii* strains

**Fig. 6:**
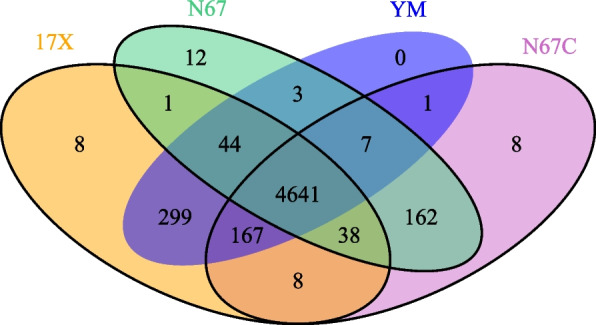
Common and strain-specific orthogroups among four *Plasmodium yoelii* strains. Venn diagram showing overlapping and strain-specific benchmarking universal single-copy orthologs (BUSCOs) among four *P. yoelii* strains**.** Details of BUSCOs and genes in each BUSCO are presented in Table S6

We next compared orthogroups and genes within the orthogroups from *P. yoelii* N67C, N67, 17X, and YM parasites (The 17X and YM sequences were downloaded from PlasmoDB (https://plasmodb.org/plasmo/app). There are 5,681, 5,749, 6,041, and 5,631 predicted genes from N67C, N67, 17X, and YM parasites, respectively (Table 3, Table S2 and Table S3). Among the predicted genes, 99.8% of the N67C genes, 98.9% of the N67 genes, 99.6% of the 17X genes, and 99.8% of the YM genes are within 5,032, 4,908, 5,206, and 5,162 orthogroups, respectively. Among the orthogroups, 4,641 are shared among all the four parasite strains (Fig. 6 and Table S7). There are also 299 and 162 orthogroups specific to the 17X/YM and N67/N67C pairs, respectively. Additionally, there are 8, 12, 8, and 0 orthogroups specific to each of the N67C, N67, 17X, and YM parasites. Because of ~ 50 times lower subread coverage for the N67 genome than that of N67C, the strain-specific orthogroups could be due to missing N67 sequences or sequence assembly errors, which can be better addressed with higher N67 genome coverage.

**Table 3:** Numbers of genes and orthogroups within four *Plasmodium yoelii* parasites

Parasite	N67C	N67	17X	YM
Total genes	5,681	5,749	6,041	5,631
No. orthogroups	5,032	4,908	5,206	5,162
No. genes in orthogroups	5,670	5,685	6,019	5,617
% genes in orthogroups	99.8	98.9	99.6	99.8
No. strain-specific orthogroups	8	12	8	0
No. genes in strain-specific orthogroups	48	37	58	0

Despite being subspecies, the 17X and N67C parasites had 557 genes with identical sequences (Table S2). GO (gene ontology) -term enrichment analysis of the homologous *P. falciparum* genes (analysis not available for *P. yoelii*) using PANTHER (https://geneontology.org/) showed top enriched terms of heterochromatin organization (GO:0070828), mitochondrial membrane organization (GO:0007006), vacuolar acidification (GO:0007035), cellular response to unfolded protein (GO:0034620), proton motive force-driven ATP synthesis (GO:0015986), rRNA 3'-end processing (GO:0031125), monoatomic ion homeostasis (GO:0050801), and purine ribonucleoside triphosphate metabolic process (GO:0009205) (Table S8). These highly conserved GO biological processes play essential roles in parasite survival and development, which can be considered as disease control targets.

### Comparison of gene families between *P. yoelii* strains

There are multiple gene families in the *P. yoelii* genomes with critical biological roles [31]. We reported 22 such gene families with our earlier N67 draft genome assembled using the HGAP algorithm [21, 32]. Our current study utilized Flye [22], a recent de novo assembler for single-molecule sequencing reads with an improved repeat detection algorithm. It is highly suitable for studying organisms like *P. yoelii* parasites with high repeat content in their genomes. Using the Flye-based genome assembly and annotation approach, we updated the N67 and N67C gene counts associated with 22 families that may be associated with virulence and host-parasite interactions (Table S9). For instance, our previous assembly using HGAP included an inflated count of 83 putative copies of a homologous protein of *P. chabaudi* erythrocyte membrane antigen 1 (*pcema1*), whereas our current N67 and N67C assemblies include 1–2 copies of the homolog of *pcema1* genes in accordance with the expected gene count of one in the most recent 17X and YM genomes (https://plasmodb.org/plasmo/app).

## Discussion

This study reports the assembled and annotated genome of the *P. yoelii* N67C parasite and an improved version of our N67 assembly from 2021, along with comparative genomics analysis of the *P. yoelii* N67C, N67, 17X, and YM parasites. Nineteen contigs totaling 22,453,501 bp were assembled de novo, establishing the sequences of the parasite's 14 chromosomes and the mitochondrial and apicoplast genomes. There were also three contigs (contigs 12, 20, and 22) that had a total of 85,644 bp (0.38%) not being assembled into any chromosomes, which suggests potential missing pieces likely residing in the polymorphic subtelomeric regions. MAKER-based gene predictions resulted in 5,681 N67C genes with good statistical support (99.9% of predicted genes having AED values smaller than 0.5). We also updated the N67 parasite genome assembly and improved the annotation of the N67 parasite genome by a ~ 7% increase in the total gene count (5383 to 5749).

Comparing the N67 and N67C parasite genomes assembled from PacBio Sequel reads with a mean coverage of 239x—664 × and 98%—100% Apicomplexa-specific BUSCO completeness, we identified 208 putative polymorphic sites, including 133 SNPs and 75 indels. These results align with previous studies suggesting that N67 and N67C are isogenic parasites derived from a recent common ancestor [14, 15]. In the previous microarray hybridization analysis, 22 and 56 SNPs were detected between N67/N67 and YM/17XNL pairs, respectively, in probe sequences that covered approximately 2% of the genome [14, 15]. The SNP rate was approximately one SNP per 20 kb (11,000 probes × 40 bp = 440 kb sequence covered by the array probes. 440 kb divided by 22 SNPs = 20 kb/SNP) between N67 and N67C, which appears to be higher than the SNP rate obtained in the current study (23,000 kb genome divided by 133 SNPs $\approx$ 173 kb per SNP). Similarly, 440 kb/56 SNPs $\approx$ 7.9 kb/SNP between YM and 17XNL by array probe analysis, whereas 92 SNPs were detected between the 17X and 17XNL genomes [12]. Considering both YM and 17XNL were derived from 17X [11] and assuming a similar mutation rate, we can estimate 92 × 2 = 184 SNPs between YM and 17XNL, which gives approximately 125 kb per SNP between YM and 17XNL. In both cases, the SNP rates by the array were much higher than those from genome sequence comparisons (8.7 times for N67 and N67C and 15.8 times for YM and 17XNL). One explanation for the higher SNP rates for the array estimates is that the microarray probes were designed based on known polymorphic sites between N67 and 17XNL sequences.

Interestingly, the SNPs and indels on the chromosomes did not appear to be randomly distributed, with chromosomes 1, 5, 7, 8, 9, 12, and 13 having fewer than 10 SNPs and indels, whereas chromosomes 2, 11, and 14 having more than 20 polymorphic sites. Additionally, there was no correlation between the numbers of SNPs and indels; for example, chromosome 9 had zero SNPs and nine indels, and chromosome 12 had six SNPs and zero indels. These polymorphic sites could occur by random mutation and were retained by functional selections. Random mutations are expected to occur more or less evenly throughout the chromosomes, with larger chromosomes having more polymorphic sites. Among the SNPs leading to amino acid changes, the S114N substitution is interesting. This polymorphism is equivalent to the S106N substitution in the *P. yoelii* 17Xpyr and the S108N substitution in *P. falciparum* Dd2 [33]. Our in vivo drug tests confirmed that this S114N substitution confer resistance to PYR. Indeed, the N67C parasite was a parasite obtained initially from MR4 (BEI, https://www.beiresources.org/About/MR4Home.aspx) under the name of *P. yoelii* 33X(Pr3) (MRA-754, deposited by Dr. David Walliker) that was selected with PYR previously [14]. In addition to conferring drug resistance, a single amino acid substitution in the parasite genome can also change disease severity and host immune responses. The C-Y (N67C^C^—N67^Y^) substitution at the amino acid position of 741 (C741Y) in the erythrocyte binding-like (PyEBL) protein modified iRBC surface and host immune responses [20]. Similarly, a C713R substitution in the same PyEBL protein trafficking domain increased parasite growth and virulence in the 17X lineage [34, 35]. In PbA, a nonsynonymous nucleotide substitution (T-C) at position 5468 (leading to an F to S amino acid substitution) in the first DNA binding domain of the ApiAP2 transcription factor gene also altered the development of host immunity [36]. Functional characterization of the detected polymorphisms may reveal their roles in parasite development and virulence.

Our RNA-Seq analysis results showed that most of the genes in the N67 and N67C parasites were expressed at similar levels. Among the 5,178 genes detected, 406 genes (7.8%) had differential expression levels at twofold or higher between the two parasites. Most of these DEGs (85%) were gene families encoding YIR and fam-a/b/c proteins, suggesting that these proteins could play an important role in parasite biology and disease phenotypes. YIR proteins are expressed on the surface of RBCs infected with late-stage asexual parasites, and host immunity can modulate *yir* gene transcription [37]. Different *yir* genes were shown to be active at various stages of the life cycle of rodent malaria parasites and may have distinct functions during parasite development [38, 39]. Additionally, activating a single *yir* gene can change the expression of many other *yir* genes [40]. The differences in *yir* gene expression between N67 and N67C likely contribute to the variation in disease phenotype and parasite growth in mice. Whether the *yir* gene expression differences in the N67 and N67C are modulated by host immune response or by specific gene expression regulation is not clear. However, some highly differentially expressed genes could be caused by the absence of specific *yir* genes in one of the parasites. Indeed, many orthogroups are subspecies-specific (17X/YM and N67/N67C pairs) or strain-specific. An orthogroup is a set of genes from multiple species descended from a single gene in the last common ancestor of that set of species [41]. Again, these subspecies- and strain-specific orthogroups largely belong to the multigene families *yir*, *fam-a*, *fam-b*, and *fam-c*. However, we cannot rule out that some of the observations in the variation in the existence and expression level of the multi-copy genes were due to sequence alignment errors.

In addition to the DEGs encoding YIR and fam proteins, the 5.4-fold (log_2_ value = 2.437) higher gene expression level encoding a putative RuvB-like helicase 1 in N67C is interesting. The *P. falciparum* RuvB1 was found to be localized mainly to the nuclear region of the parasite and contain both ATPase and DNA helicase activities translocating in 5′ to 3′ direction [42]. This protein could be involved in the DNA and RNA metabolism of the parasites, which may affect the availability of parasite nucleic acids that interact with the host immune system and, therefore, influence host IFN-I responses. Several genes encoding DNA/RNA binding proteins or enzymes in nucleoside metabolism may also affect host recognition of parasite nucleic acids, leading to changes in host IFN-I responses. With the genome-wide polymorphisms and DEGs identified from the N67 and N67C parasites, functional characterization of candidate genes having mutations or DEGs between the two parasites may lead to a better understanding of the molecular mechanisms of malaria pathogenesis.

## Conclusion

The rodent malaria parasites N67C and N67 are important strains of *P. y. nigeriensis* subspecies that produce very different disease phenotypes. The molecular mechanisms and the parasite genes contributing to the difference in parasite biology, virulence, and pathology remain largely unknown. This study sequences, assembles, and annotates the genome of the N67C parasites and compares the N67C genome and transcriptomes with those of N67 published previously [21] and other *P. yoelii* subspecies, revealing candidate polymorphic sites and DEGs for future functional characterization.

## Methods

### Parasites, mice, and ethics statement

The N67C [previously under the name of *P. y. yoelii* 33X(Pr3)] parasite was initially obtained from MR4-BEI (https://www.beiresources.org/About/MR4.aspx) [14]. Inbred female C57BL/6j mice, aged 6–8 weeks old, were obtained from the NIAID/Taconic repository. The procedures for infection of mice with the parasites and blood collection were reported previously [21, 43] and were performed in accordance with the protocol approved (approval #LMVR11E) by the Institutional Animal Care and Use Committee (IACUC) at the National Institute of Allergy and Infectious Diseases (NIAID). Parasitemia was monitored by counting Giemsa-stained thin blood smears under a microscope after intraperitoneal (IP) injection of 1 × 10^6^ parasites in 100 ml sterile PBS.

### Parasite DNA and RNA sample preparation

Blood samples (200 ml) with approximately 30 ~ 40% parasitemia containing similar proportions of blood-stage parasites (ring, trophozoite, and schizont) were collected on day 4 after IP injection of mice with 1 × 10^6^ iRBCs. The procedure to prepare DNA for PacBio sequencing was as described previously [21]. Briefly, iRBCs in 1 ml of 1.5% sodium citrate/0.9% sodium chloride buffer were centrifuged at 500 g for 5 min, resuspended in 1 ml PBS, and passed through two consecutive NWF filters (Zhixing Bio, Bengbu, China) to remove white blood cells [44]. The cells were then washed in 800 ml PBS 3 times, lyzed in 150 ml lysis buffer (pH8.0) containing 100 mM NaCl, 10 mM Tris, 25 mM EDTA, 0.5% SDS, 4 ml RNase A solution (500 mg/ml), and 20 ml proteinase K solution (10 mg/ml). The lysate was incubated at room temperature for 30 min, and DNA was extracted using a Qiagen MegAttract HMW DNA kit. The extracted DNA was run on 1% agarose gel to confirm the presence of a typical high molecular weight band.

For cDNA sequencing, iRBCs after NWF filter treatment were placed in RNA-later, and total RNAs were extracted using the Direct-zol™ RNA MiniPrep (Zymo Research) following the manufacturer's protocol. Five hundred nanograms of the total RNA was used to prepare the sequencing library using Illumina TruSeq Stranded mRNA library prep kits.

### PacBio DNA sequencing

The standard PacBio library preparation procedure (Pacific Biosciences, Menlo Park, CA, USA) was performed as described previously [21]. Briefly, the N67C parasite genomic DNA isolated from the blood asexual stages was used to generate DNA libraries for sequencing. Genomic DNA was fragmented by mechanical shearing, and DNA fragments to > 20 kb were purified. The fragments were then tailed with an A-overhang and ligated with T-overhang SMRTbell adapters. Sequencing primer and Sequel DNA polymerase were annealed and bound to the SMRTbell Library, respectively. SMRT sequencing was performed on a Sequel System with Sequel Sequencing Kit 3.0, 1,200 min movies. The default SMRT Link QC pipeline was used in the quality control (QC) analysis of raw reads (subreads).

### Genome assembly

We used Flye v2.9 [22] for assembling the N67C genome and improving our N67 genome from 2021, which had been assembled using HGAP v4.0. The Flye v2.9 has the repeat graph approach that utilizes approximate sequence matches (instead of exact k-mer matches) while assembling the repeat-rich *Plasmodium yoelii* genome using noise-prone single-molecule sequencing (SMS) reads. Upon using the highest Apicomplexa-specific BUSCO criterion to select the Flye assembly settings across multiple assemblies, the N67C genome assembly was generated using the first setting with raw reads (Flye –pacbio-raw mode with the subreads.fastq input) and the second setting with ccs reads (–pacbio-hifi mode with the ccs.fastq input, –genome-size of 23.5 m, –asm-coverage of 200, and –min-overlap of 10,000). On the other hand, the N67 Flye assembly settings were –pacbio-raw mode with the subreads.fastq input, –genome-size of 23.5 m, and –asm-coverage of 200. The resulting contigs were scaffolded using RagTag v2.0.1 [45, 46] with default settings utilizing the *P. y. yoelii 17X* genome reference (PlasmoDB release 67 and 68, accessed in February and May 2024, respectively: https://plasmodb.org/plasmo/app).

### Illumina RNA sequencing (RNA-Seq) and quality control analysis

RNA samples from five mice infected with N67 and five infected with N67C were sequenced using the Illumina Sequencing method. Adapter sequences were removed using Trimmomatic [47]. The quality of the input RNA and next-generation sequencing (NGS) libraries was evaluated using Bioanalyzers and Nanodrop. Data was processed following the rna-seek workflow (https://github.com/OpenOmics/RNA-seek), which trimmed raw fastq files using Cutadapt v4.7 [48], mapped reads using STAR v2.7.10b in 2-pass mode [49], evaluated data quality using RSeQC [24], Picard [25], and FastQC [26], and generated raw counts using RSEM (in gtf/gif formats) [27]. Statistical differential expression was performed using DESeq2 [50]”.

### Gene model predictions

N67C and N67-specific gene predictions were generated using the MAKER pipeline [28]. MAKER utilized BLAST to align the 17X transcripts and proteins to the de novo assembled genome, polished these alignments using Exonerate in a splice-aware fashion and implemented SNAP and Augustus hidden Markov models (HMMs) to generate gene models [51, 52]. Functional analysis and annotation were performed with InterProScan [53] after homology searches of over 15 databases, including Pfam, ProSite, TIGRFAM, and PANTHER. Our final sets of N67C and N67 genes and proteins only include those adequately supported by the assembled genomes in conjunction with the 17X proteome. For each predicted protein, we computed two AED values (AED/eAED: at the base pair and exon levels) to quantify how well each protein is supported by these data sources [AED] [54].

### Estimates of genome completeness

The completeness of the de novo assembled N67C transcriptome, genome, and proteome was evaluated using BUSCO (Benchmarking Universal Single-Copy Orthologs) [23, 55] against the single-copy orthologs conserved among Apicomplexa (446 BUSCOs) and *Plasmodium* (3,642 BUSCOs) from the OrthoDB v10.1 database (https://v10-1.orthodb.org/) [56].

### Identification of orthologs

Ortholog sets from the 17X, YM, N67, and N67C parasites were identified using Orthofinder [41, 57]. DIAMOND [58] was used for identifying sequence similarity, and DendroBLAST [59] was used in gene tree inference.

### Polymorphic sites identification and confirmation

Polymorphic sites between N67 and N67C genome sequences were detected after mapping the PacBio CCS reads to the N67C assembly using pbmm2 v1.13.1 (https://github.com/PacificBiosciences/pbmm2). Variants were called using DeepVariant [60], and a joint VCF (variant call format) file was generated using glnexus v1.4.1 [61]. The resulting variants were filtered using a threshold ≥ Q20 and annotated to determine the effect of the variants on genes using VEP 107 [62]. To confirm selected polymorphisms between N67 and N67C, primers flanking polymorphic sites (see Table S6 for primer sequences) were designed to amplify DNA sequences from the N67 and N67C parasites. PCR reaction in a 15 μl volume contained 10 μ1 master PCR mix, 3 μl DNA solution (∼50 pg), and 1 μl forward and reverse primer solution (2 μM) each. The master PCR mix contained 0.5 μl Taq DNA polymerase (1 U/l), 0.3 μl dNTP solution (10 mM), 2.4 μl MgCl_2_ (25 mM), and 1.5 μl 10X PCR buffer. The cycling program included 94 °C for 2 min for initial denaturation, followed by 94 °C for 20 s, 50 to 55 °C for 20 s, 60 °C for 30 s for 40 cycles, and a final extension at 60 °C for 2 min. PCR products were separated on 1—2% agarose gels. The PCR products were sequenced by a commercial company (Quintara Biosciences, San Francisco, CA).

## Supplementary Information

Supplementary Material 1: Table S1. Quality control statistics of RNA-Seq samples from mixed blood stages of Plasmodium yoelii nigeriensis N67 and N67C parasites.

Supplementary Material 2: Table S2. Predicted Plasmodium yoelii nigeriensis N67C gene and protein sequences with quality statistics and orthologs of P. y. yoelii 17X, P. y. yoelii 17X, P. y. nigeriensis N67, and Plasmodium falciparum.

Supplementary Material 3: Table S3. Predicted Plasmodium yoelii nigeriensis N67 gene and protein sequences with quality statistics and orthologs of P. y. yoelii 17X, P. y. yoelii YM, P. y. nigeriensis N67C, and Plasmodium falciparum.

Supplementary Material 4: Table S4. Differentially expressed single-copy genes (DEGs) between Plasmodium yoelii nigeriensis N67 and N67C.

Supplementary Material 5: Table S5. Polymorphic sites between the Plasmodium yoelii nigeriensis N67C and N67 parasites with corresponding IDs and predicted gene functions for the four P. yoelii parasites: N67C, N67, 17X, and YM.

Supplementary Material 6: Table S6. Verification of the putative nucleotide polymorphisms between Plasmodium yoelii nigeriensis N67 and N67C genomes through PCR amplification and sequencing.

Supplementary Material 7: Table S7. Annotated ortholog groups among the four Plasmodium yoelii parasites (17X, YM, N67C, and N67).

Supplementary Material 8: Table S8. GO-term enrichment of genes that are 100% conserved between Plasmodium yoelii nigerinesis N67C and P. y. yoelii 17X.

Supplementary Material 9: Table S9. Predicted gene families and copy numbers in four Plasmodium yoelii parasites: P. y. yoelii 17X, P. y. yoelii YM, P. y. nigeriensis N67, and P. y. nigeriensis N67C.

## Data Availability

The N67C and N67 genome sequences have been deposited to GenBank (https://www.ncbi.nlm.nih.gov/biosample?Db=biosample&DbFrom=bioproject&Cmd=Link&LinkName=bioproject_biosample&LinkReadableName=BioSample&ordinalpos=1&IdsFromResult=694977) with the BioSample IDs: SAMN41801232; Sample name: N67_gDNA_2024; BioSample: SAMN41801231; Sample name: N67C_gDNA_2024; BioSample: SAMN17587902; Sample name: bc1115. The RNAseq raw fastq file and counts are available at GEO (https://www.ncbi.nlm.nih.gov/geo/query/acc.cgi?acc=GSE278246) under accession # GSE278246.

## Abbreviations

YM: *Plasmodium y.* *yoelii YM*
17X: *Plasmodium y.* *yoelii 17X*
17XNL: *Plasmodium y. **yoelii* 17XNL
17XL: *Plasmodium y. **yoelii* 17XL
N67: *Plasmodium y. **nigeriensis* N67
N67C: *Plasmodium y. **nigeriensis* N67C
IFN-I: Type I interferon
PyEBL: *P.**yoelii* Erythrocyte binding-like
SNPs: Single nucleotide polymorphisms
AED: Annotation edit distance
Pi: Post-infection
BUSCO: Benchmarking universal single-copy orthologs
ISGs: Interferon-stimulated genes
Yir: *Yoelii* Interspersed repeats
iRBCs: Infected-red blood cells
QC: Quality control
HMMs: Hidden Markov models
LCB: Locally collinear block
DEG: Differentially expressed genes
DHFR-TS: Dihydrofolate reductase-thymidylate synthase
QTL: Quantitative trait loci
RNA-Seq: RNA sequencing
EST: Expressed sequence tag
FDR: False discovery rate
PYR: Pyrimethamine
GO: Gene ontology
